# Structure, Function, and Regulation of the SRMS Tyrosine Kinase

**DOI:** 10.3390/ijms21124233

**Published:** 2020-06-14

**Authors:** Chakia J. McClendon, W. Todd Miller

**Affiliations:** 1 Department of Physiology and Biophysics, Stony Brook University, Stony Brook, NY 11794-8661, USA; chakia.mcclendon@stonybrook.edu; 2 Department of Veterans Affairs Medical Center, Northport, NY 11768, USA

**Keywords:** tyrosine kinase, signal transduction, phosphorylation, SH3 domains, SH2 domains, Src family kinases

## Abstract

Src-related kinase lacking C-terminal regulatory tyrosine and N-terminal myristoylation sites (SRMS) is a tyrosine kinase that was discovered in 1994. It is a member of a family of nonreceptor tyrosine kinases that also includes Brk (PTK6) and Frk. Compared with other tyrosine kinases, there is relatively little information about the structure, function, and regulation of SRMS. In this review, we summarize the current state of knowledge regarding SRMS, including recent results aimed at identifying downstream signaling partners. We also present a structural model for the enzyme and discuss the potential involvement of SRMS in cancer cell signaling.

## 1. Introduction

Src-related kinase lacking C-terminal regulatory tyrosine and N-terminal myristoylation sites (SRMS) is a 53 kDa nonreceptor tyrosine kinase that was first discovered in mouse neural precursor cells [1]. Studies of the expression pattern of SRMS mRNA showed that the highest levels were present in lung, testes, and liver tissues. Full-length cDNA encoding SRMS was isolated from a murine lung cDNA library, with the predicted SRMS protein containing all of the conserved amino acid residues expected for a nonreceptor tyrosine kinase. Targeted disruption of the gene encoding SRMS did not lead to any apparent phenotype, suggesting that the kinase may have functions that are redundant in regard to other tyrosine kinases [1]. A few years later, the SRMS gene (designated PTK70) was isolated from a neonatal murine skin library and was found to be most strongly expressed in normal epidermal and keratinocyte cells [2].

While SRMS was initially considered to be a member of the Src family of nonreceptor tyrosine kinases, analyses of the amino acid sequence and intron/exon structure placed SRMS in a separate family, the Brk family [3,4]. This group of nonreceptor tyrosine kinases also includes the Fyn-related kinase (Frk) and breast tumor kinase (Brk; also called PTK6) [3,4,5]. The human SRMS gene maps to chromosome 20q13.3, which is only 1.5 kbp away from the Brk gene, suggesting that the two genes may interact genetically [4,6]. The two genes also map together on chromosome 2 in mice [1,4]. Indeed, the two protein kinases interact biochemically; SRMS phosphorylates the C-terminus of Brk [7]. This portion of human chromosome 20 is a region of the genome that was found to be amplified in breast cancers [8]. SRMS, like Brk, is highly expressed in most breast carcinoma cells compared to normal mammary cell lines and tissues [9].

The goal of this review is to summarize past and recent findings regarding SRMS. Compared to other nonreceptor tyrosine kinases, there is very little information available regarding the structure, function, and regulation of SRMS. Herein, we review what is known about the substrates and downstream signaling partners of SRMS. Wherever possible, we compare SRMS to the well-characterized nonreceptor tyrosine kinase Src. Our goal is to provide a foundation for future studies in the field.

## 2. Structure and Activity of SRMS

### 2.1. Domain Architecture

**Figure 1:**
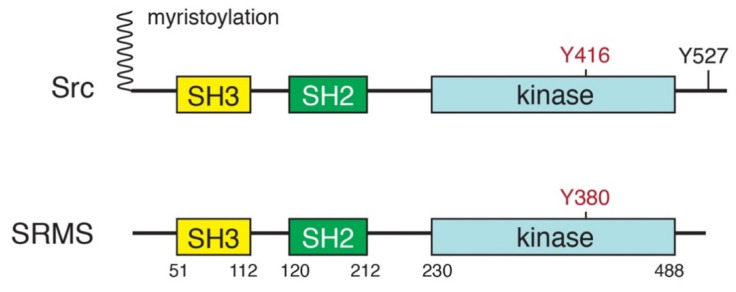
Domain structures of Src kinase and SRMS (Src-related kinase lacking C-terminal regulatory tyrosine and N-terminal myristoylation sites). The major autophosphorylation sites are indicated in red. In Src, phosphorylation of Y527 stabilizes the autoinhibited conformation. SRMS lacks the N-terminal myristoylation site found in Src.

SRMS is a 488-amino acid nonreceptor tyrosine kinase containing three functional domains that was first identified in Src family kinases (SFKs). From N-terminus to C-terminus, these are the Src homology 3 (SH3), Src homology 2 (SH2), and kinase catalytic domains [1,3] (Figure 1). Within the SFKs, this domain architecture is critical for mediating inter- and intramolecular interactions, downstream signaling, and regulation [10,11,12,13]. The SH3–SH2-kinase domain arrangement is found in other Brk family kinases, as well as in the Src, Abl, Tec, and Csk families of nonreceptor tyrosine kinases, and was conserved from unicellular eukaryotes to humans [14,15,16]. Brk family kinases appear to be absent from the genomes of premetazoans. Brk-like kinases are found in *Amphioxus* and the cnidarian *Nematostella vectensis*, suggesting that an SFK/Brk gene in a metazoan ancestor was duplicated, giving rise to SFKs and Brk-family kinases [17]. SRMS itself is present only in vertebrates.

**Figure 2:**
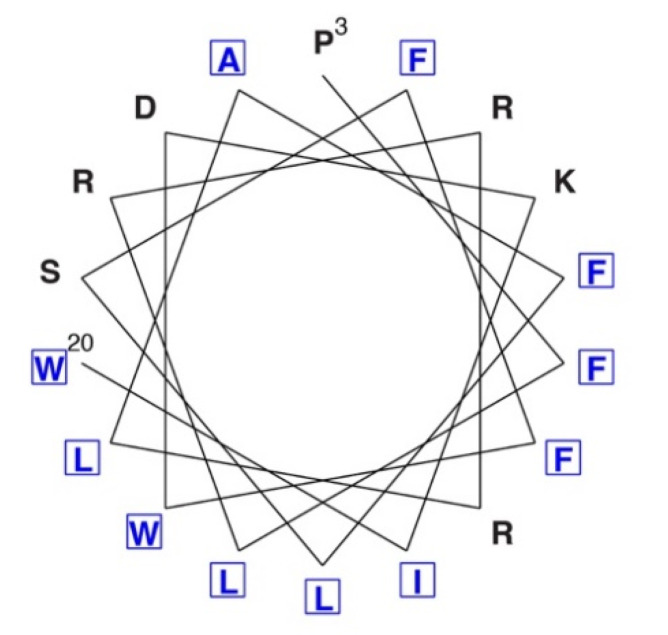
N-terminal portion of the SRMS unique domain. Residues Pro3–Trp20 were plotted on a helical wheel diagram using PEPWHEEL (www.bioinformatics.nl/cgi-bin/emboss/pepwheel). Hydrophobic residues are shown in blue with boxes.

Src contains a 14-carbon bound myristoyl group on the N-terminus that is capable of interacting with the inner leaflet of the plasma membrane [10,18,19,20]. Protein N-myristoylation is a co- and post-translational modification that requires removal of the N-terminal Met residue, and enzymatic myristoyl transfer to a conserved Gly residue. Myristoylation of Src allows the kinase to interact with a variety of extracellular receptors and to activate a variety of downstream signaling pathways that trigger cell transformation [19,21]. Myristoylation is important for the role of Src in prostate cancer development [22]. Unlike Src, SRMS lacks the myristoylation site and membrane anchoring signals (Figure 1). Therefore, SRMS is an intracellular protein (discussed in more detail below). The amino terminal region of SRMS contains a 51-amino acid sequence preceding the SH3 domain. In Src family kinases, this region is unique to each kinase and is involved in specific protein–protein interactions with signaling partners [23]. NMR and small-angle x-ray scattering (SAXS) experiments showed that an intrinsically disordered region within the c-Src unique domain forms a fuzzy intramolecular complex involving the SH3 domain [24]. For SRMS, removal of the N-terminal region results in a complete loss of kinase activity in transfected mammalian cells, indicating that the N-terminus plays an important role in maintaining kinase activity [9]. The N-terminal portion of the SRMS unique domain has the potential to form an amphipathic (or amphiphilic) alpha helix (Figure 2). These structures are often important in mediating protein–membrane or intermolecular interactions that regulate activity [25]. The unique domain of Frk shares this feature, suggesting that the N-termini of these two Brk family kinases might play similar roles. The C-terminal portion of the SRMS unique domain is proline-rich, which is consistent with the hypothesis that intrinsically disordered domains engage in intramolecular interactions with adjacent SH3 domains [26]. Such an interaction could help to stabilize the active conformation of SRMS, thereby explaining the essential role of the N-terminus in activity.

SH3 and SH2 domains are structurally and functionally conserved modules found in a wide variety of signaling proteins [27,28]. They function by mediating protein–protein interactions, where SH3 domains bind to proline-rich sequences within polyproline type II helices, and SH2 domains bind to phosphotyrosine-containing sequences. In SFKs, these domains have positive and negative roles in signal transduction. The positive role reflects the ability of the SH3 and SH2 domains to facilitate protein-to-protein interactions, allowing the kinase to bind and phosphorylate cellular substrates [10,29]. Their negative roles arise from intramolecular interactions that assemble SFKs into autoinhibited conformations with low catalytic activity [10,30]. Abl and Tec family kinases are also inhibited by intramolecular interactions involving the SH3 and SH2 domains [14]. These two families of nonreceptor tyrosine kinases have additional domains that play roles in enzymatic regulation. Disruption of SH3/SH2-mediated intramolecular interactions in SFKs leads to a pronounced increase in kinase activity [14,23,31,32]. For Abl, mutations within the SH3 domain lead to enzyme activation and cellular transformation, while SH2 domain deletion causes a complete loss of kinase activity [33]. We recently confirmed that the isolated SRMS SH2 domain is a functional pTyr-binding module with binding affinities comparable to the Src SH2 domain (McClendon and Miller, SH2-dependent phosphorylation of Cas by SRMS, manuscript in preparation). The importance of the SH3 and SH2 domains of SRMS was tested by expressing wild type and domain-deleted forms of the kinase in mammalian cells [9]. Deletion of the SH3 domain did not affect autophosphorylation activity, as assessed by anti-pTyr Western blotting. In contrast, deletion of the enzyme’s SH2 domain led to a significant reduction in autophosphorylation [9].

The catalytic domain of SRMS is composed of ~258 amino acids with approximately 54% amino acid identity to Src. Src contains a C-terminal tail with a conserved Tyr residue (Y527; chicken c-Src numbering). Phosphorylation of Src Y527 by another tyrosine kinase (Csk) produces an intramolecular interaction with the SH2 domain of Src, stabilizing the autoinhibited conformation [10,23]. Brk is regulated in a similar manner [7,34]. The C-terminal regulatory tail found in Brk and Src does not exist in SRMS, suggesting that the enzyme must be regulated by a different mechanism. Phosphorylation may still play an important role; 15 phosphotyrosine sites within the SH2 and kinase domains were identified via quantitative phosphoproteomics [35].

### 2.2. Structure of SRMS Kinase

**Figure 3:**
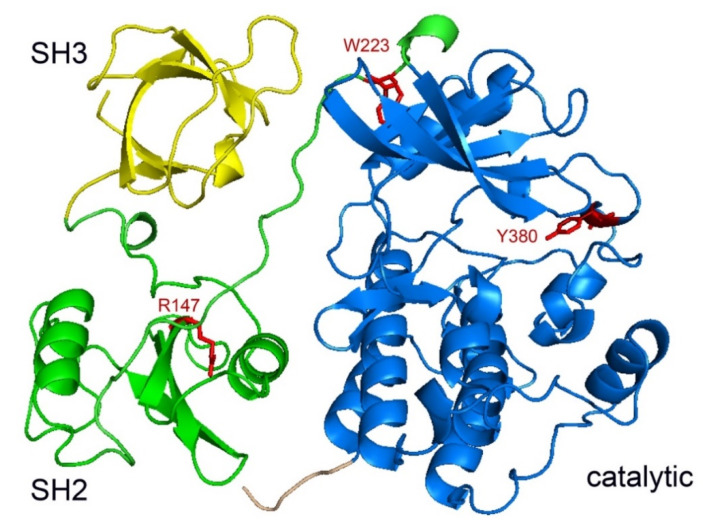
Model of the SH3–SH2-kinase domains of SRMS. The three-dimensional structure of human SRMS was modeled on the known structure of chicken c-Src (Protein Data Bank pdb code: 2H8H) using the homology detection program HHpred and MODELLER v. 9.22 from the Max Planck Institute Bioinformatics Toolkit [37,38].

There is currently no three-dimensional structure of SRMS kinase. We created a model of the SH3–SH2-kinase domains of SRMS using a crystallographic structure of Src in a complex with a quinazoline inhibitor (pdb code = 2H8H) [36]. The high degree of homology with Src (approximately 42% for SH3–SH2-kinase) [3] indicates that the overall architecture of SRMS is likely to be well conserved (Figure 3). On the other hand, the unique N-terminal region of SRMS, which plays an important role in enzyme regulation [9], is divergent from the Src N-terminus and was not included in the modeling. SRMS contains a tyrosine residue (Y380) that is homologous with Src Y416, the major autophosphorylation site [1]. Mutation of SRMS Y380 to Phe reduced the phosphorylation of SRMS in human embryonic kidney (HEK) 293T cells, suggesting that this residue is also the major autophosphorylation site in SRMS [9]. In the model, Y380 is present in the putative activation loop of SRMS and the phenolic sidechain of Y380 is pointed toward the active site in a position compatible with autophosphorylation (Figure 3).

R147 of SRMS is in the enzyme’s SH2 domain and corresponds to R175 of Src, a conserved Arg residue in the phosphotyrosine recognition pocket that makes critical electrostatic interactions with pTyr [27]. In the structure of autoinhibited Src-family kinases, R175 interacts with the phosphorylated C-terminal tyrosine (Src Y527) to stabilize the inhibited conformation [30,39]. An R147A mutation was not shown to affect SRMS autophosphorylation activity in mammalian cells [9]. SRMS lacks the C-terminal tyrosine and the tail sequence is predicted to be too short to reach the phosphotyrosine recognition pocket of the SRMS SH2 domain (Figure 3). On the other hand, the relative positioning of the SH2 and catalytic domains of SRMS is compatible with the ability of the enzyme to progressively phosphorylate substrates containing nine or more pTyr residues away from the phosphorylatable tyrosine (McClendon and Miller, SH2-dependent phosphorylation of Cas by SRMS, manuscript in preparation). 

Mutation of W223 to alanine abrogated SRMS kinase activity [9]. A tryptophan residue is conserved at this position in Src-family kinases and plays an important regulatory role. In Src kinases, mutation of the corresponding tryptophan (W260) destabilizes the SH3–kinase interaction and increases enzyme activity [40]. Similarly to Src, W223 is predicted to lie near the SH3–kinase interface of SMRS (Figure 3). In addition to the essential role of the N-terminal region [9], this interface is presumed to be intact for stabilization of the active form of SRMS.

### 2.3. In Vitro Activity of SRMS

Our laboratory previously reported the expression and purification of full-length murine SRMS from *Spodoptera frugiperda* (Sf9) insect cells using a recombinant baculovirus vector [7]. The purified enzyme demonstrated tyrosine kinase activity, as measured by a continuous spectrophotometric assay or by transfer of ^32^P from labeled ATP to synthetic substrates. SRMS displayed the expected velocity vs. [enzyme] relationship up to at least 500 nM SRMS, although the specific activity was significantly lower than that of Src. SRMS was inactive toward synthetic peptides containing recognition sequences for other tyrosine kinases (e.g., Src, Abl, epidermal growth factor receptor, insulin receptor). In vitro activity was only detectable against the random copolymer, poly(Glu, Tyr) (4:1); in this respect, it resembles Csk kinase [7]. SRMS and Csk share a number of structural features. They both contain the three functional domains (SH3, SH2, and kinase), but lack the N-terminal myristoylation site and C-regulatory tail site; they are therefore incapable of SH2-dependent autoinhibition. Csk function is regulated by binding to the transmembrane phosphoprotein Cbp/PAG1 and recruitment to the plasma membrane [41]. No analogous regulatory protein was identified for SRMS.

SRMS was inhibited by the Src/Abl ATP-competitive inhibitor dasatinib, but not by the small molecule kinase inhibitors imatinib, nilotinib, sorafenib, VX680, or BIRB796 [7]. Other small molecules that showed activity against SRMS include a pyrazolopyridine compound [42] and a pyrrolo pyrimidine [43], both of which would be predicted to bind at the ATP site. Both of these inhibitors are also active against the Src family kinase Lck, suggesting that the SRMS active site may share features with this enzyme. An unexplored avenue for inhibitor development is the disruption of interactions involving the essential N-terminal region.

## 3. Substrates of SRMS

Src family kinase-mediated phosphorylation of cellular substrates is important for mitosis, cell spreading, adhesion, motility, cell death, survival, and differentiation [23,44,45]. In many cases, recognition of key substrates by SFKs is well characterized. In contrast, relatively few physiological substrates are reported for SRMS. In the following section, we discuss the current identified substrates of SRMS. The majority of these candidate substrates were identified through important proteomic experiments carried out by Lukong and colleagues [35,46].

The first bona fide SRMS substrate to be identified was docking protein 1 (Dok1). Dok1 is an adaptor protein that is a negative regulator of several signaling pathways and can act as a tumor suppressor. For example, Dok1 protein expression is lost in gastric tumor cells compared to normal gastric tissue [47]. Dok1 serves as a substrate for many tyrosine kinases, including SFKs, and was initially identified as a potential SRMS substrate in a proteomic study [48]. Dok1 and SRMS were subsequently shown to interact in HEK293T cells. SRMS binds to Dok1 via its SH3 and SH2 domains; in the latter case, binding requires prior phosphorylation of Dok1 [9]. Expression of SRMS increases Dok1 tyrosine phosphorylation, and in vitro reactions confirmed that Dok1 is a direct substrate of the kinase [9]. SRMS-mediated phosphorylation might modulate the function of Dok1, as observed in cases of other tyrosine kinases [49,50].

Brk kinase is another protein that was demonstrated to serve as a direct substrate for SRMS. In contrast to Src family kinases, the C-terminal inhibitory tyrosine of Brk (Y447) is not phosphorylated by Csk [7]. Given the close linkage of Brk and SRMS on human chromosome 20, the two kinases were co-expressed in Src/Yes/Fyn triple knock-out fibroblasts to examine a possible physical interaction. Brk (or a kinase-dead version) was phosphorylated upon SRMS co-expression. Mass spectrometry experiments identified Brk Y447 as the primary site of SRMS-mediated phosphorylation on BRK [7]. SRMS-mediated phosphorylation of Y447 did not directly inhibit Brk, however; it appears that SRMS acts in tandem with protein tyrosine phosphatase 1B to modulate Brk [7].

Goel et al. used proteomic analyses to pinpoint prospective binding partners of SRMS. A label-free quantitative phosphoproteomic study was performed to profile tyrosine-phosphorylated proteins in HEK293 cells expressing SRMS [35]. Total cell proteins were trypsin-digested, and tyrosine-phosphorylated peptides were enriched by immunoaffinity purification. LC-MS/MS results revealed over 1100 tyrosine-phosphorylated peptides in SRMS-overexpressing cells. These peptides were derived from 663 proteins with roles in RNA processing, cell cycle, protein ubiquitination, and signal transduction [35]. Importantly, peptides derived from Dok1 and the Y380 autophosphorylation site of SRMS were identified in lysates from SRMS-expressing cells, validating previous findings. Motif analyses of the tyrosine phosphorylated peptides indicated that many sequences contained a lysine residue at the −2 or −4 position N-terminal to tyrosine. A different set of primary sequence determinants for SRMS was identified using a peptide library approach, i.e., XIYX and YXXV, where X = any amino acid [51].

As a follow-up to the proteomic experiments, immobilized peptide arrays were used with lysates from SRMS-expressing HEK293 cells to confirm phosphorylation of a subset of the peptides. Based on these findings, the authors carried out directed experiments to confirm that two proteins, vimentin and Sam68, are direct substrates of SRMS [35]. Vimentin is an intermediate filament protein that is modified by serine/threonine and tyrosine phosphorylation, as well as numerous other post-translational modifications [52]. Phosphorylation of vimentin is believed to promote the protein’s pro-migratory properties. Mice lacking vimentin displayed impaired wound healing due to defects in fibroblast migration [53]. Src associated in mitosis 68 (Sam68) is a major RNA-binding protein implicated in cellular RNA-metabolic processes such as mRNA splicing and stability [54]. Overexpression of Sam68 mammalian cells inhibits cell cycle progression and cell proliferation and induced apoptosis [54]. Interestingly, Sam68 was the first in vivo substrate identified for the closely related Brk kinase [55]. Tyrosine phosphorylation of Sam68 by Brk reduces the ability of Sam68 to bind RNA. SRMS could similarly be involved in the regulation of Sam68; SRMS was found to be critical in the epidermal growth factor (EGF) stimulated phosphorylation of Sam68 [35].

A second proteomic study from the Lukong laboratory took a complementary approach, specifically, the authors used TiO_2_-based enrichment of peptides from SRMS-expressing HEK293T cell lysates [46]. This study focused on the identification of pSer/pThr-containing proteins, i.e., not direct SRMS substrates, but rather proteins whose phosphorylation was indirectly regulated by expression of SRMS. Sixty SRMS-dependent phosphoproteins were identified, and analysis of the phosphorylation motifs identified 25 candidate Ser/Thr kinases that could lie downstream of SRMS. Casein kinase 2 alpha was predicted to target the largest number of phosphorylation sites in the SRMS-dependent phosphoproteome, suggesting that this kinase might represent a downstream target for SRMS regulation [46].

## 4. Subcellular Localization of SRMS

Plasma membrane localization of Src family kinases is critical to their function [10,44]. Removal of the N-terminal myristoylation signal from v-Src, the transforming oncoprotein encoded by Rous sarcoma virus, blocks its ability to transform mammalian cells in culture [19]. Abl kinase has two splice variant proteins, referred to as 1a and 1b. The latter contains a N-terminal myristoylation site similar to that in Src-family PTKs, which enables interaction with the cell membrane. However, the shorter 1a variant lacks the myristoylation modification. The lack of N-myristoylation in SRMS reduces the likelihood of plasma membrane association. Indeed, endogenous SRMS was found to localize to distinct punctate cytoplasmic structures in three breast cancer cell lines (MDA-MB-231, AU565, and SKBR3) and in the cervical cancer cell line HeLa [9]. Ectopically expressed GFP-SRMS displayed a similar punctate cytoplasmic localization. Subcellular fractionation experiments in MDA-MB-231, HeLa, and HBL-100 cells confirmed that SRMS was localized in the cytoplasm and not in the nucleus or plasma membrane [9]. These findings suggest that the presence or absence of N-terminal myristoylation is a major distinguishing feature in the function of Src family kinases vs. SRMS. The cytoplasmic localization of SRMS contrasts with the closely related Brk kinase, which is found both in the nucleus and cytoplasm in a variety of cell types. Brk is nuclear in normal prostate epithelial cells but cytoplasmic in poorly differentiated tumors, suggesting that its localization may play a role in oncogenic signaling [56].

The individual domains of SRMS play varying roles in directing subcellular localization [9]. GFP-SRMS was found in punctate cytoplasmic structures in over 90% of transfected HEK293 cells. In contrast, for a GFP-tagged variant lacking the N-terminal region, approximately 30% of the cells showed localization in these puncta, with the remaining 70% giving a diffuse cytoplasmic localization. The N-terminal region contains the putative amphipathic alpha helical region (Figure 2), which could mediate self-association or formation of liquid-separated phases, potentially contributing to the observed punctate structures. In the case of c-Src, the N-terminal region plays a role in clustering (although, in this case, membrane localization is also an important component) [57,58,59].

A mutant lacking the SH3 domain localized to puncta in 80% of cells, while a mutant lacking the SH2 domain exhibited a diffuse localization pattern in 90% of the transfected cells. A kinase-dead mutant of SRMS (K258M) showed a diffuse localization pattern of over 80% of transfected cells, implying that SRMS activity has a significant impact on subcellular localization. For example, based on the results with the SH2-deleted mutant, SRMS-mediated phosphorylation could produce a stable interaction with substrates residing in the punctate structures. In this way, the SRMS-substrate complex could generate a spatially localized signal. The identification of SRMS substrates provided additional clues to the intracellular localization of the kinase. In some cases, SRMS substrates co-localize with specific cytoplasmic structures. Two such potential substrates are Sam68 and vimentin; confocal microscopy experiments confirmed that SRMS co-localizes with these proteins within the cytoplasm of HEK293 cells. In the case of vimentin, SRMS was found in intermediate filaments rather than the cytoplasmic puncta, suggesting that vimentin recruits SRMS to these structures [35]. Dok1 and SRMS were shown to be co-expressed in several breast cancer cell lines, including HBL100 [9]. On the other hand, no significant co-localization with SRMS was observed; Dok1 was predominantly nuclear in these cells, with a small amount of cytoplasmic localization. Thus, the interaction between Dok1 and SRMS might be regulated rather than constitutive. Phosphorylation of Dok1 by SRMS might sequester Dok1 in the cytoplasm to promote downstream signaling.

## 5. Function of SRMS in Normal Cells

**Figure 4:**
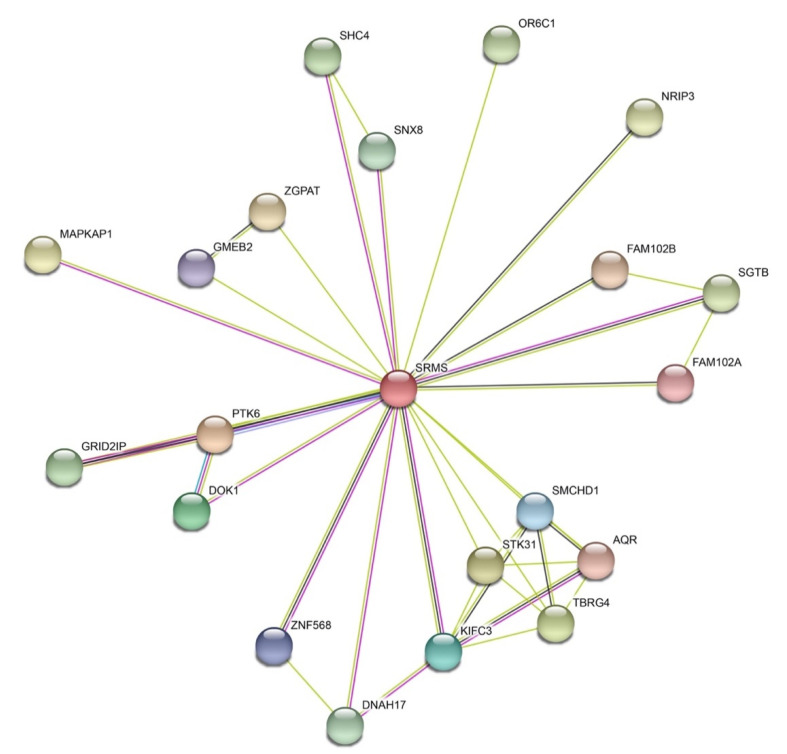
The SRMS connectome. The human SRMS connectome was analyzed using STRING, version 11 [62]. Data represent 1st shell with a minimum required interaction score of 0.4 and a maximum number of interactors of 20 (https://string-db.org). Blue lines represent known interactions from curated databases, magenta lines represent experimentally determined interactions, light green lines represent predicted interactions from textmining, and black lines represent predicted interactions from co-expression.

The understanding of SRMS function is still in its very early stages, particularly compared to the well-studied Src family kinases. Functional characterizations of the other Brk family kinases (Frk and Brk) are even further described. Numerous substrates for these kinases were identified, with both growth-promoting and growth-suppressing signaling roles depending on the cell and tissue context [4,5]. Brk is expressed primarily in epithelial cells within the gastrointestinal tract, skin, and prostate, where it plays a role in cell differentiation. SRMS is expressed widely in normal mammalian tissue samples and in several cancer cell lines. No apparent defects were present in SRMS-deficient mice, suggesting that SRMS might have roles that are normally redundant with other tyrosine kinases [1]. Knockdown of SRMS expression with siRNA was used to shed light on the normal functions of the kinase. In MDA-MB-231 cells, SRMS was shown to be necessary for EGF-stimulated tyrosine phosphorylation of Sam68 [35]. Knockdown of SRMS in U2OS human osteosarcoma cells led to a depletion of LC3 (microtubule-associated protein 1 light chain 3 alpha), indicative of an increase in autophagic flux. Thus, SRMS could play a role as an inhibitor of autophagy [60]. As noted above, the identification of SRMS substrates provided important clues to the function of the kinase. Using bioinformatics approaches, the available proteomic data was recently incorporated into an interaction database to elucidate the SRMS signaling network [61]. We performed a STRING analysis [62] to identify protein–protein interactions involving SRMS (Figure 4). The known and predicted SRMS interactions show potential connections to a number of other signaling proteins, including Ser/Thr kinase 31, sorting nexin 8, SHC-transforming protein 4, and the target of rapamycin complex 2 subunit MAPKAP1 (Figure 4).

## 6. Function of SRMS in Human Disease

At present, SRMS is not linked conclusively to any human disease state. The possibility that SRMS is overexpressed or dysregulated in various malignancies was explored previously, showing that SRMS was overexpressed in six of eight breast cancer cell lines tested, exhibiting the highest levels of expression in HBL-100 and the lowest expression in MDA-MB-468 and AU565 cells [9]. SRMS expression was much lower in the normal mammary cell line 184B5. Using immunohistochemistry, Lukong and colleagues showed strong expression of SRMS in human invasive ductal carcinomas compared to adjacent normal breast tissue [9]. Furthermore, the levels of SRMS expression correlated with the grade and severity of the tumor. The other members of the Brk family also play a role in the development and progression of breast cancer. Brk was originally discovered in a screen for tyrosine kinases expressed in human metastatic breast tumors [63]. Brk expression was low or undetectable in normal mammary tissues or in benign lesions, but overexpressed in a significant fraction of breast tumors [64]. Brk is co-amplified and co-expressed with the receptor tyrosine kinase ErbB2/HER2 in human breast cancers and cooperates with ErbB2 to stimulate downstream signaling [65].

The involvement of SRMS in other cancers was also studied. In a recent proteomic study of serum samples from gastric cancer patients, SRMS was the only kinase found to be differentially expressed in cancer samples compared to normal controls [66]. Machida et al. [67] profiled lysates from human lung cancer cell lines by measuring binding of their tyrosine-phosphorylated proteins to a series of SH2 domain probes. The SRMS SH2 domain showed significant binding in a number of the cell lines. Moreover, higher levels of SRMS SH2 binding correlated with cell lines harboring mutant forms of epidermal growth factor receptor (EGFR), cell lines that were more sensitive to the EGFR inhibitor erlotinib, and cells in which EGFR and MET receptors were both activated [67]. A bioinformatics analysis of phosphopeptides from clear cell renal cell carcinoma identified SRMS as a potential upstream kinase [68].

Numerous copy number gains and missense mutations involving SRMS were identified in breast cancer and other cancers (e.g., [69]), but the functional effects are not presently characterized. In addition to mutations that activate or inactivate a tyrosine kinase, mutations can alter kinase signaling pathways or “rewire” the signaling network downstream of a particular kinase. Using a bioinformatics approach, Creixell et al. analyzed cancer somatic mutations to identify those with the highest potential to rewire downstream signaling. For SRMS, the somatic mutations most likely to display altered specificity were (in order of decreasing specificity score) D378N, Y299H, A353T, T302M, F230L, E418K, V366A, W249L, A394V, E396D/K, P467T, V409A, A474T, and A256V [70]. The effects of these mutations on SRMS function await experimental testing.

## 7. Summary

The contribution of SRMS to normal cellular function has yet to be firmly established. It is particularly important to confirm the identities of additional upstream and downstream signaling partners and substrates of SRMS and to assess their impact on cellular phenotypes. Because of the lack of a clear phenotype in SRMS-deficient mice, it may be necessary to produce cellular or animal models in which SRMS is silenced along with other nonreceptor tyrosine kinases to eliminate redundancy. In particular, it is not clear whether SRMS plays unique or overlapping roles with other Brk family kinases (and/or Src family kinases) and whether any differences might be manifested in a cell- or tissue-specific manner. It is also not yet known whether SRMS functions as a bona fide driver in cancer cells. Additional studies are warranted in order to clarify the functions of SRMS and to determine whether SRMS and its signaling networks represent potential drug targets for cancer therapy.

## Abbreviations

ATP	Adenosine triphosphate
Brk	Breast tumor kinase
Dok1	Docking protein 1
EGF	Epidermal growth factor
Frk	Fyn-related kinase
GFP	Green fluorescent protein
HEK	Human embryonic kidney
LC-MS	Liquid chromatography-mass spectrometry
PTK6	Protein tyrosine kinase 6
SFK	Src family kinase
SH2	Src homology 2
SH3	Src homology 3
siRNA	Small interfering RNA
SRMS	Src-related kinase lacking C-terminal regulatory tyrosine and N-terminal myristoylation sites

## References

[1] Kohmura N., Yagi T., Tomooka Y., Oyanagi M., Kominami R., Takeda N., Chiba J., Ikawa Y., Aizawa S. (1994). A novel nonreceptor tyrosine kinase, Srm: Cloning and targeted disruption. Mol. Cell Biol..

[2] Kawachi Y., Nakauchi H., Otsuka F. (1997). Isolation of a cDNA encoding a tyrosine kinase expressed in murine skin. Exp. Dermatol..

[3] Serfas M.S., Tyner A.L. (2003). Brk, Srm, Frk, and Src42A form a distinct family of intracellular Src-like tyrosine kinases. Oncol. Res..

[4] Brauer P.M., Tyner A.L. (2010). Building a better understanding of the intracellular tyrosine kinase PTK6—BRK by BRK. Biochim. Biophys. Acta.

[5] Ostrander J.H., Daniel A.R., Lange C.A. (2010). Brk/PTK6 signaling in normal and cancer cell models. Curr. Opin. Pharmacol..

[6] Park S.H., Lee K.H., Kim H., Lee S.T. (1997). Assignment of the human PTK6 gene encoding a non-receptor protein tyrosine kinase to 20q13.3 by fluorescence in situ hybridization. Cytogenet. Cell Genet..

[7] Fan G., Aleem S., Yang M., Miller W.T., Tonks N.K. (2015). Protein-tyrosine phosphatase and kinase specificity in regulation of SRC and breast tumor kinase. J. Biol. Chem..

[8] Hodgson J.G., Chin K., Collins C., Gray J.W. (2003). Genome amplification of chromosome 20 in breast cancer. Breast Cancer Res. Treat..

[9] Goel R.K., Miah S., Black K., Kalra N., Dai C., Lukong K.E. (2013). The unique N-terminal region of SRMS regulates enzymatic activity and phosphorylation of its novel substrate docking protein 1. FEBS J..

[10] Brown M.T., Cooper J.A. (1996). Regulation, substrates and functions of src. Biochim. Biophys. Acta.

[11] Mayer B.J. (1997). Signal transduction: Clamping down on Src activity. Curr. Biol..

[12] Cooper J.A., Howell B. (1993). The when and how of Src regulation. Cell.

[13] Yadav S.S., Miller W.T. (2008). The evolutionarily conserved arrangement of domains in SRC family kinases is important for substrate recognition. Biochemistry.

[14] Shah N.H., Amacher J.F., Nocka L.M., Kuriyan J. (2018). The Src module: An ancient scaffold in the evolution of cytoplasmic tyrosine kinases. Crit. Rev. Biochem. Mol. Biol..

[15] Suga H., Miller W.T. (2018). Src signaling in a low-complexity unicellular kinome. Sci. Rep..

[16] Suga H., Torruella G., Burger G., Brown M.W., Ruiz-Trillo I. (2014). Earliest Holozoan expansion of phosphotyrosine signaling. Mol. Biol. Evol..

[17] D’Aniello S., Irimia M., Maeso I., Pascual-Anaya J., Jimenez-Delgado S., Bertrand S., Garcia-Fernandez J. (2008). Gene expansion and retention leads to a diverse tyrosine kinase superfamily in amphioxus. Mol. Biol. Evol..

[18] Buss J.E., Kamps M.P., Gould K., Sefton B.M. (1986). The absence of myristic acid decreases membrane binding of p60src but does not affect tyrosine protein kinase activity. J. Virol..

[19] Kamps M.P., Buss J.E., Sefton B.M. (1986). Rous sarcoma virus transforming protein lacking myristic acid phosphorylates known polypeptide substrates without inducing transformation. Cell.

[20] Roskoski R. (2015). Src protein-tyrosine kinase structure, mechanism, and small molecule inhibitors. Pharm. Res..

[21] Bromann P.A., Korkaya H., Courtneidge S.A. (2004). The interplay between Src family kinases and receptor tyrosine kinases. Oncogene.

[22] Kim S., Alsaidan O.A., Goodwin O., Li Q., Sulejmani E., Han Z., Bai A., Albers T., Beharry Z., Zheng Y.G. (2017). Blocking myristoylation of Src inhibits its kinase activity and suppresses prostate cancer progression. Cancer Res..

[23] Parsons S.J., Parsons J.T. (2004). Src family kinases, key regulators of signal transduction. Oncogene.

[24] Arbesu M., Maffei M., Cordeiro T.N., Teixeira J.M., Perez Y., Bernado P., Roche S., Pons M. (2017). The unique domain forms a fuzzy intramolecular complex in Src family kinases. Structure.

[25] Kaiser E.T., Kezdy F.J. (1983). Secondary structures of proteins and peptides in amphiphilic environments. (A review). Proc. Natl. Acad. Sci. USA.

[26] Arbesu M., Pons M. (2019). Integrating disorder in globular multidomain proteins: Fuzzy sensors and the role of SH3 domains. Arch. Biochem. Biophys..

[27] Kuriyan J., Cowburn D. (1997). Modular peptide recognition domains in eukaryotic signaling. Annu. Rev. Biophys. Biomol. Struct..

[28] Mayer B.J. (2015). The discovery of modular binding domains: Building blocks of cell signalling. Nat. Rev. Mol. Cell Biol..

[29] Miller W.T. (2003). Determinants of substrate recognition in nonreceptor tyrosine kinases. Acc. Chem. Res..

[30] Sicheri F., Kuriyan J. (1997). Structures of Src-family tyrosine kinases. Curr. Opin. Struct. Biol..

[31] Briggs S.D., Sharkey M., Stevenson M., Smithgall T.E. (1997). SH3-mediated Hck tyrosine kinase activation and fibroblast transformation by the Nef protein of HIV-1. J. Biol. Chem..

[32] Moarefi I., LaFevre-Bernt M., Sicheri F., Huse M., Lee C.H., Kuriyan J., Miller W.T. (1997). Activation of the Src-family tyrosine kinase Hck by SH3 domain displacement. Nature.

[33] Mayer B.J., Baltimore D. (1994). Mutagenic analysis of the roles of SH2 and SH3 domains in regulation of the Abl tyrosine kinase. Mol. Cell Biol..

[34] Qiu H., Miller W.T. (2002). Regulation of the nonreceptor tyrosine kinase brk by autophosphorylation and by autoinhibition. J. Biol. Chem..

[35] Goel R.K., Paczkowska M., Reimand J., Napper S., Lukong K.E. (2018). phosphoproteomics analysis identifies novel candidate substrates of the nonreceptor tyrosine kinase, Src-related kinase lacking C-terminal regulatory tyrosine and N-terminal myristoylation sites (SRMS). Mol. Cell Proteom..

[36] Hennequin L.F., Allen J., Breed J., Curwen J., Fennell M., Green T.P., Lambert-van der Brempt C., Morgentin R., Norman R.A., Olivier A. (2006). N-(5-chloro-1,3-benzodioxol-4-yl)-7-[2-(4-methylpiperazin-1-yl)ethoxy]-5- (tetrahydro-2H-pyran-4-yloxy)quinazolin-4-amine, a novel, highly selective, orally available, dual-specific c-Src/Abl kinase inhibitor. J. Med. Chem..

[37] Webb B., Sali A. (2016). Comparative protein structure modeling using MODELLER. Curr. Protoc. Protein Sci..

[38] Zimmermann L., Stephens A., Nam S.Z., Rau D., Kubler J., Lozajic M., Gabler F., Soding J., Lupas A.N., Alva V. (2018). A completely reimplemented MPI bioinformatics toolkit with a new HHpred server at its core. J. Mol. Biol..

[39] Xu W., Harrison S.C., Eck M.J. (1997). Three-dimensional structure of the tyrosine kinase c-Src [see comments]. Nature.

[40] LaFevre-Bernt M., Sicheri F., Pico A., Porter M., Kuriyan J., Miller W.T. (1998). Intramolecular regulatory interactions in the Src family kinase Hck probed by mutagenesis of a conserved tryptophan residue. J. Biol. Chem..

[41] Okada M. (2012). Regulation of the SRC family kinases by Csk. Int. J. Biol. Sci..

[42] Wenglowsky S., Ahrendt K.A., Buckmelter A.J., Feng B., Gloor S.L., Gradl S., Grina J., Hansen J.D., Laird E.R., Lunghofer P. (2011). Pyrazolopyridine inhibitors of B-RafV600E. Part 2: Structure-activity relationships. Bioorg. Med. Chem. Lett..

[43] Anastassiadis T., Deacon S.W., Devarajan K., Ma H., Peterson J.R. (2011). Comprehensive assay of kinase catalytic activity reveals features of kinase inhibitor selectivity. Nat. Biotechnol..

[44] Thomas S.M., Brugge J.S. (1997). Cellular functions regulated by Src family kinases. Annu. Rev. Cell Dev. Biol..

[45] Schlessinger J. (2000). New roles for Src kinases in control of cell survival and angiogenesis. Cell.

[46] Goel R.K., Meyer M., Paczkowska M., Reimand J., Vizeacoumar F., Vizeacoumar F., Lam T.T., Lukong K.E. (2018). Global phosphoproteomic analysis identifies SRMS-regulated secondary signaling intermediates. Proteome Sci..

[47] Li T., Li B., Sara A., Ay C., Leung W.Y., Zhang Y., Dong Y., Liang Q., Zhang X., Weidner P. (2019). Docking protein-1 promotes inflammatory macrophage signaling in gastric cancer. Oncoimmunology.

[48] Takeda H., Kawamura Y., Miura A., Mori M., Wakamatsu A., Yamamoto J., Isogai T., Matsumoto M., Nakayama K.I., Natsume T. (2010). Comparative analysis of human SRC-family kinase substrate specificity in vitro. J. Proteome Res..

[49] Noguchi T., Matozaki T., Inagaki K., Tsuda M., Fukunaga K., Kitamura Y., Kitamura T., Shii K., Yamanashi Y., Kasuga M. (1999). Tyrosine phosphorylation of p62(Dok) induced by cell adhesion and insulin: Possible role in cell migration. EMBO J..

[50] Liang X., Wisniewski D., Strife A., Shivakrupa, Clarkson B., Resh M.D. (2002). Phosphatidylinositol 3-kinase and Src family kinases are required for phosphorylation and membrane recruitment of Dok-1 in c-Kit signaling. J. Biol. Chem..

[51] Deng Y., Alicea-Velazquez N.L., Bannwarth L., Lehtonen S.I., Boggon T.J., Cheng H.C., Hytonen V.P., Turk B.E. (2014). Global analysis of human nonreceptor tyrosine kinase specificity using high-density peptide microarrays. J. Proteome Res..

[52] Snider N.T., Omary M.B. (2014). Post-translational modifications of intermediate filament proteins: Mechanisms and functions. Nat. Rev. Mol. Cell Biol..

[53] Ivaska J., Pallari H.M., Nevo J., Eriksson J.E. (2007). Novel functions of vimentin in cell adhesion, migration, and signaling. Exp. Cell Res..

[54] Taylor S.J., Resnick R.J., Shalloway D. (2004). Sam68 exerts separable effects on cell cycle progression and apoptosis. BMC Cell Biol..

[55] Derry J.J., Richard S., Carvajal H.V., Ye X., Vasioukhin V., Cochrane A.W., Chen T., Tyner A.L. (2000). Sik (BRK) phosphorylates Sam68 in the nucleus and negatively regulates its RNA binding ability. Mol. Cell Biol..

[56] Derry J.J., Prins G.S., Ray V., Tyner A.L. (2003). Altered localization and activity of the intracellular tyrosine kinase BRK/Sik in prostate tumor cells. Oncogene.

[57] Le Roux A.L., Busquets M.A., Sagues F., Pons M. (2016). Kinetics characterization of c-Src binding to lipid membranes: Switching from labile to persistent binding. Colloids Surf. B Biointerfaces.

[58] Owen D.M., Rentero C., Rossy J., Magenau A., Williamson D., Rodriguez M., Gaus K. (2010). PALM imaging and cluster analysis of protein heterogeneity at the cell surface. J. Biophotonics.

[59] Smith A.W., Huang H.H., Endres N.F., Rhodes C., Groves J.T. (2016). Dynamic organization of myristoylated Src in the live cell plasma membrane. J. Phys. Chem. B.

[60] Potts M.B., Kim H.S., Fisher K.W., Hu Y., Carrasco Y.P., Bulut G.B., Ou Y.H., Herrera-Herrera M.L., Cubillos F., Mendiratta S. (2013). Using functional signature ontology (FUSION) to identify mechanisms of action for natural products. Sci. Signal..

[61] Buffard M., Naldi A., Radulescu O., Coopman P.J., Larive R.M., Freiss G. (2019). Network reconstruction and significant pathway extraction using phosphoproteomic data from cancer cells. Proteomics.

[62] Szklarczyk D., Gable A.L., Lyon D., Junge A., Wyder S., Huerta-Cepas J., Simonovic M., Doncheva N.T., Morris J.H., Bork P. (2019). STRING v11: Protein-protein association networks with increased coverage, supporting functional discovery in genome-wide experimental datasets. Nucleic Acids Res..

[63] Mitchell P.J., Barker K.T., Martindale J.E., Kamalati T., Lowe P.N., Page M.J., Gusterson B.A., Crompton M.R. (1994). Cloning and characterisation of cDNAs encoding a novel non-receptor tyrosine kinase, brk, expressed in human breast tumours. Oncogene.

[64] Barker K.T., Jackson L.E., Crompton M.R. (1997). BRK tyrosine kinase expression in a high proportion of human breast carcinomas. Oncogene.

[65] Xiang B., Chatti K., Qiu H., Lakshmi B., Krasnitz A., Hicks J., Yu M., Miller W.T., Muthuswamy S.K. (2008). Brk is coamplified with ErbB2 to promote proliferation in breast cancer. Proc. Natl. Acad. Sci. USA.

[66] Yoo M.W., Park J., Han H.S., Yun Y.M., Kang J.W., Choi D.Y., Lee J.W., Jung J.H., Lee K.Y., Kim K.P. (2017). Discovery of gastric cancer specific biomarkers by the application of serum proteomics. Proteomics.

[67] Machida K., Eschrich S., Li J., Bai Y., Koomen J., Mayer B.J., Haura E.B. (2010). Characterizing tyrosine phosphorylation signaling in lung cancer using SH2 profiling. PLoS ONE.

[68] Ghosh A.P., Willey C.D., Anderson J.C., Welaya K., Chen D., Mehta A., Ghatalia P., Madan A., Naik G., Sudarshan S. (2017). Kinomic profiling identifies focal adhesion kinase 1 as a therapeutic target in advanced clear cell renal cell carcinoma. Oncotarget.

[69] Stephens P.J., Tarpey P.S., Davies H., Van Loo P., Greenman C., Wedge D.C., Nik-Zainal S., Martin S., Varela I., Bignell G.R. (2012). The landscape of cancer genes and mutational processes in breast cancer. Nature.

[70] Creixell P., Schoof E.M., Simpson C.D., Longden J., Miller C.J., Lou H.J., Perryman L., Cox T.R., Zivanovic N., Palmeri A. (2015). Kinome-wide decoding of network-attacking mutations rewiring cancer signaling. Cell.

